# Associations Between Adherence to the Mediterranean Diet and Incident Sarcopenia in Prospective Cohort Studies

**DOI:** 10.3390/nu17020313

**Published:** 2025-01-16

**Authors:** Ligia J. Dominguez, Nicola Veronese, Lee Smith, Francesco Saverio Ragusa, Piero Schirò, Giovanna Di Bella, Mario Barbagallo

**Affiliations:** 1 Department of Medicine and Surgery, “Kore” University of Enna, 94100 Enna, Italy; 2 Geriatric Unit, Department of Internal Medicine and Geriatrics, University of Palermo, 90127 Palermo, Italy; nicola.veronese@unipa.it (N.V.); francescosaverioragusa@gmail.com (F.S.R.); giovannadibella79@libero.it (G.D.B.); mario.barbagallo@unipa.it (M.B.); 3 Center for Health, Performance and Wellbeing, Anglia Ruskin University, Cambridge CB1 1PT, UK; lee.smith@aru.ac.uk; 4 Primary Care Department, Provincial Health Authority (ASP) of Palermo, 90100 Palermo, Italy; pierosch@hotmail.it

**Keywords:** mediterranean diet, sarcopenia, frailty, aging, muscle, prospective, cohort

## Abstract

The loss of skeletal muscle mass and strength, known as sarcopenia, is prevalent in older adults and linked to an increased risk of disability, frailty, and early mortality. Muscle health is crucial for the functionality and independence of older adults. As the aging population continuously grows, finding cost-effective strategies for preventing and treating sarcopenia is an important public health priority. While nutrition is recognized as a key factor in the development of sarcopenia, its role in preventing and treating the condition is still under investigation. In recent decades, nutritional research has shifted from a focus on individual nutrients or healthy foods to examining the combination of nutrients and foods in dietary patterns, along with their potential synergistic and antagonistic effects. A balanced diet and regular participation in physical activity are essential for maintaining musculoskeletal health. One of the healthy eating patterns with the greatest evidence of multiple health benefits is the Mediterranean diet, which has also been linked to positive effects on muscle function in observational studies. However, there is a lack of intervention studies. This review explores the updated evidence from longitudinal prospective studies on associations between adherence to the Mediterranean diet and sarcopenia in order to promote preventive and intervention strategies for healthy muscle aging.

## 1. Introduction

The primary challenges to achieving a healthy lifespan are age-related physiological decline in various organs, leading to functional limitations, disability, and diminished quality of life, while also increasing the risk and severity of age-related chronic diseases [1]. One of the most significant age-related changes is the shift in body composition, marked by a decrease in skeletal muscle mass and an increase in body fat [2]. These changes typically begin in mid-adulthood and gradually progress over time. After the age of 60, there is an estimated annual loss of 3% of muscle mass, accompanied by a preferential loss of fast-twitch type II myofibers, which are converted into slower type I fibers. This is further compounded by the denervation of motor units and fat infiltration within and between the fibers [3,4]. Although the loss of muscle strength and power with advancing age is even more pronounced [5], longitudinal studies indicate substantial variability in muscle mass and strength decline among older adults [6,7,8]. This variability underscores the potential importance of modifiable factors, such as nutrition and physical activity, in influencing muscle aging [9,10].

Sarcopenia is a muscle condition characterized by reduced muscle mass, strength, and quality [11,12], and is linked to functional decline, lower quality of life, cognitive deterioration, and increased mortality [13,14,15]. Since October 2016, sarcopenia has had an assigned code (M62.84) in the International Classification of Disease, Tenth Revision, Clinical Modification (ICD-10-CM) [16]. The introduction of the new ICD-10-CM code for sarcopenia marks a significant advancement in acknowledging sarcopenia as a medical condition. However, not all countries have adopted this ICD-10 CM code, and the definition of sarcopenia is highly variable. A recent study comparing data obtained with twelve different definitions of sarcopenia found a prevalence ranging from 0.7% to 16.8% in community-dwelling participants aged over 70 years from four European countries [17]. In the UK, sarcopenia affected 4.6% to 7.9% older adults in the Hertfordshire Cohort Study depending on gender and the definition used [18]. More recent data from the UK Biobank reported an overall prevalence of 5.3% among community-dwelling participants aged 40–70 years, which increased with age and comorbidity [19]. In Italy, a study reported remarkably high prevalence of sarcopenia among older adults admitted to hospital, reaching 34.7% [20]. The prevalence of sarcopenia was also high (31.6% of women and 17.4% of men) in a sample of Italian community-dwelling older people aged 80 years or older [21].

The precise causes of sarcopenia remain complex and not fully understood. A combination of factors, such as malnutrition [22,23,24], physical inactivity [13,24], and age-related chronic diseases [25,26], contribute to the progressive weakening of muscle function with age. A large number of observational and intervention studies have focused on individual nutrients to explore the connection between diet and muscle health in aging (e.g., protein or vitamin D, with or without exercise) [27,28,29,30]. However, isolating the effect of a single dietary component from others and understanding its interactions within the context of a whole diet on health outcomes is difficult. Recently, studies adopting a whole-diet approach have emerged to investigate the role of dietary patterns in muscle health during aging [31,32,33]. This approach recognizes the complexity of human dietary habits in relation to health [34,35,36] and the challenge of understanding the effects of nutrients and bioactive components within an entire diet when assessing their impact on aging muscles. Nevertheless, since nutrition can impact the development of sarcopenia, such a prevalent condition associated with aging, this topic warrants further exploration.

From this perspective, it is crucial to determine which nutritional approach is most beneficial for preventing and managing sarcopenia, as people consume combinations of foods, not individual nutrients. Numerous prospective observational studies and trials across diverse populations have confirmed the positive impact of greater adherence to the Mediterranean diet in preventing and managing age-related non-communicable diseases, including cardiovascular and metabolic conditions, neurodegenerative diseases, cancer, depression, respiratory issues, and fragility fractures [37,38]. There are also several population-based cross-sectional and longitudinal studies exploring the impact of the Mediterranean diet on skeletal muscle health (including mass, strength, function, physical performance, and sarcopenia) in older adults [32,39,40,41].

The aim of the present review is to summarize and update the available evidence of the relationship between adherence to the Mediterranean diet and incident sarcopenia and its components in longitudinal prospective studies. First, we will describe the origin, components, and consumption recommendations of the Mediterranean diet. We will then describe the concept of sarcopenia and its different definitions. Finally, we will discuss the available evidence on the relationship between adherence to the Mediterranean diet and the incidence of sarcopenia.

## 2. Mediterranean Diet: Concept, Origin, and Components

The concept of the Mediterranean diet, as we understand it today, was first introduced through the Seven Countries Study, led by Keys and colleagues in the 1950s [42]. This ecological study, primarily focused on impoverished rural populations in the isle of Crete (Greece) and southern Italy, laid the groundwork for the hypothesis that diet may have protective health benefits, such as reducing the risk of cardiovascular diseases and cancer [37]. It is important to note that the Mediterranean diet, initially made known by Keys and colleagues in the twentieth century, reflects the dietary habits and lifestyle that Mediterranean populations have followed for thousands of years. The Mediterranean region has unique culinary traditions, rich in local products, aromas, flavors, and vibrant colors. These traditions emphasize taste, as well as connection with nature and a deep respect for it, while also placing great importance on the preparation, sharing, and enjoyment of food with family and friends [43,44].

**Figure 1:**
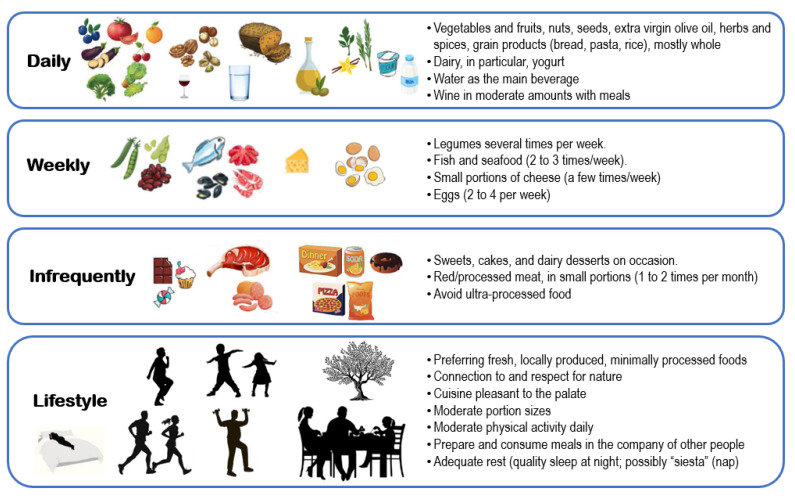
Nutritional and lifestyle characteristics of the Mediterranean diet.

The traditional Mediterranean diet is characterized by high consumption of vegetables, fruits, legumes, whole grains, and nuts; generous consumption of healthy fats from olive oil, commonly used in cooked vegetables, legumes, and salads; moderate-to-high consumption of sea products (fish and shellfish); and moderate consumption of fermented dairy products [37,45]. Consuming large amounts of fruits and vegetables, which are low in calories but rich in nutrients and fiber, supports long-term adherence to this healthy and high-quality diet, ensuring nutritional adequacy without the need for taking supplements [46]. The inclusion of extra virgin olive oil and various aromatic herbs enhances the flavor of plant-based dishes, making it easier to maintain a high intake of fresh, flavorful vegetables [47]. The traditional Mediterranean diet emphasizes two crucial elements: a high ratio of monounsaturated to saturated fats, primarily due to the use of olive oil as the main or sole cooking fat, and high consumption of unprocessed or minimally processed plant-based foods, including fruits, vegetables, nuts, and legumes [45]. Figure 1 illustrates the recommended daily/weekly consumption for foods and combinations contained in the Mediterranean diet and non-nutritional components of this high-quality dietary and lifestyle model based on references [37,43,45]. It includes the consumption of a variety of fresh vegetables and fruits two to three times a day; two to four tablespoons of olive oil daily, serving as the primary (and almost sole) source of dietary fat for seasoning and cooking; and nuts and seeds as snacks, preferably daily. The consumption of legumes is recommended several times per week. Whole grains should be consumed daily, and fish and seafood should be eaten two to three times a week. Dairy, particularly yogurt, is encouraged, with small portions of cheese consumed less frequently. Eggs are included in the diet at a rate of two to four per week. Herbs and spices are used regularly to season meals. Sweets, as well as red and processed meats, should be eaten infrequently, typically once a week or less. Water is the main beverage used in the Mediterranean diet, while wine may be consumed in small amounts, always with meals if not contradictory to religious and social norms or health conditions. Components of the Mediterranean diet not directly related to its nutrient composition include connection to and respect for nature; moderate portions; moderate physical activity daily; and preparing and consuming meals in the company of other people (e.g., family and friends). It also comprises having adequate rest (sufficient and quality sleep at night; possibly short periods of rest during the day as a “siesta” = nap); preference for fresh, locally produced foods that have undergone minimal industrial processes; and cuisine pleasant to the palate [37,43,45].

The traditional Mediterranean diet is not low in fat; instead, it features a relatively high fat intake, primarily from monounsaturated fats found in olive oil and tree nuts, while maintaining a low intake of saturated fats. Additionally, following the Mediterranean diet involves a significant reduction (or even elimination) in the consumption of processed meats, red meats, sugary desserts, and, most notably, ultra-processed foods that are high in added sugars, low-quality fats, and additives, all of which have been observed to have deleterious effects on health [45,48,49,50].

In general, the health benefits of the Mediterranean diet are likely the result of synergistic interactions among its various components as a whole dietary pattern, rather than the isolated effects of individual food groups [37,45]. However, it also calls for a minimal intake of meat (especially processed meats), meat products, and ultra-processed foods.

The beneficial effects of the Mediterranean diet are primarily linked to mechanisms such as a reduction in inflammatory and oxidative stress markers, improvements in the lipid profile, insulin sensitivity, and endothelial function, as well as antithrombotic and antiatherosclerotic properties. These effects are largely attributed to the polyphenol content in the diet [51,52]. The impact of these phenolic compounds, including hydroxytyrosol, tyrosol, oleocanthal, resveratrol, and many other bioactive dietary phenols, has been extensively studied. These compounds have demonstrated significant antioxidant and anti-inflammatory effects [51,52]. Indeed, the Mediterranean diet is characterized by a low Dietary Inflammatory Index, highlighting its potential anti-inflammatory effects [53]. This dietary model positively influences the gut microbiota and immune system in diverse conditions, including emerging evidence supporting its effectiveness against severe acute respiratory syndrome coronavirus 2 [54].

From a biomolecular perspective, the Mediterranean diet exhibits various anti-inflammatory effects [55] and may influence mitochondrial processes [56]. As a result, the Mediterranean diet composition makes it well suited for preventing and managing numerous age-related medical conditions [37,57], including sarcopenia, which will be explored in the longitudinal studies discussed below.

## 3. The Concept of Sarcopenia and Its Various Definitions

The term “sarcopenia” was initially coined by Rosemberg in 1988 to refer to a decline in muscle mass, strength, and function [58]. However, there is no universally accepted definition of sarcopenia, as different international working groups dedicated to the condition have proposed varying definitions over time. This necessarily creates emerging difficulties in data comparison and unification when diverse definitions of sarcopenia are applied. The most commonly used definitions are those proposed by the following research groups or international associations: the EWGSOP [59]; the revised version of the EWGSOP [11]; the Asian Working Group on Sarcopenia (AWGS) [60]; the revised version of the AWGS [61]; the International Working Group on Sarcopenia [62]; the Foundation of the National Institute of Health-Sarcopenia Project [63]; the Sarcopenia Definition and Outcomes Consortium [64].

**Figure 2:**
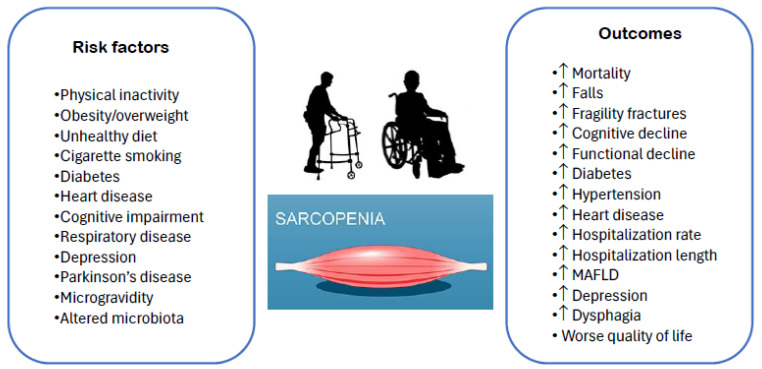
Risk factors and outcomes of sarcopenia. MAFLD: metabolic-associated fatty liver disease.

Sarcopenia is characterized by a gradual decline in muscle mass, strength, and function, which results in reduced physical performance, a higher risk of falls, fractures, functional deterioration, frailty, and mortality [11,13]. Sarcopenia also represents a significant healthcare burden, in particular regarding the estimated cost of hospitalizations in persons with sarcopenia [65] worsened by the co-presence of frailty [66]. While it primarily affects older adults, sarcopenia can also develop in mid-life [11] and is particularly common in specific populations, including persons affected by cancer [67,68], diabetes [69], liver disease [70], and kidney dysfunction [71]. Additionally, sarcopenia serves as a significant prognostic factor for survival and clinical complications in these patient groups [67,68,69,70,71]. A number of risk factors and health outcomes, such as those included in Figure 2, have been reported to be associated with sarcopenia. A recent review summarized the findings from 109 studies on the consequences and 25 studies on the risk factors of sarcopenia [72] involving patients with highly diverse characteristics. Overall, the authors found that sarcopenia was associated with an increased risk of mortality, cognitive decline, falls and fractures, hospitalization rate, functional decline, metabolic syndrome and diabetes, non-alcoholic fatty liver disease, depression, hypertension, and dysphagia. However, the risks for the diverse outcomes were highly variable considering the moderate to high heterogeneity among the studies; some associations had large confidence intervals due to the small sample sizes involved, while some others were retrospective, where the measurement error of sarcopenia made the results uncertain. Moreover, the associations might have been significantly affected by the diverse definitions of sarcopenia used and variations in muscle mass measurements, which limited the comparability of these associations [72].

Thus, the evidence hitherto is strong for few conditions. An umbrella review on the association of sarcopenia with the development of other medical conditions including six meta-analyses with 14 associations found that sarcopenia was associated with higher risk of other comorbidities and mortality in 11 of the 14 outcomes explored. However, only three outcomes, i.e., the association between sarcopenia and increased mortality risk in community-dwelling older people, disability, and falls, presented highly suggestive evidence. Other associations were classified as having only weak evidence [13].

The factors contributing to sarcopenia, falls, and fractures are multifactorial, encompassing both modifiable and non-modifiable elements [13,73]. Among the modifiable risk factors, the importance of nutrition in muscle health and sarcopenia is becoming increasingly evident. Adopting a healthy lifestyle, which includes a balanced diet and regular physical activity, avoiding sedentary behavior, such as that of the Mediterranean diet, is essential for optimizing musculoskeletal health [74,75]. Interestingly, a recent study examined the association of sarcopenia at baseline with quality of life (QoL) at a 10-year follow-up in a large representative sample of older English adults. Among 4044 older participants (mean age: 70.7 years) sarcopenia at baseline was associated with a significant five-times higher incidence of poor QoL after adjusting for several potential confounders [14].

Sarcopenia is commonly evaluated through physical measurements of total lean body mass, appendicular lean mass, or muscle mass and size. In addition, muscle strength and physical performance are used as indicators of the muscle function part of the definition of sarcopenia, and assessed through grip strength, leg muscle strength, walking speed, or a combination of physical performance tests such as walking speed, chair stands, and balance [11,62,76]. Several risk factors for sarcopenia, including smoking, diabetes, medications, and elevated body mass index (BMI), have been explored in previous studies [13,77,78]. However, suboptimal diets and poor nutritional status are prevalent among the elderly, especially in frail individuals. As a result, enhancing diet and nutrition could be an effective approach for both preventing and treating sarcopenia [79].

While muscle atrophy is a common occurrence with aging, the variability observed in this process [80] suggests that there may be factors influencing the rate at which muscle strength and function decline. Indeed, aging is associated with significant physiological changes, which may contribute to muscle loss and the development of sarcopenia. Two factors recently studied in this context are increased levels of reactive oxygen species and systemic chronic low-grade inflammation [81], which can also be linked to unhealthy diet and lack of physical activity. A study across 12 countries found that nearly two-thirds of older participants were at nutritional risk or malnourished [82]. Reduced food intake can lead to weight and muscle loss. Deficiencies in overall diet and/or specific nutrients due to inadequate food intake may be linked to the development of sarcopenia.

Preclinical and clinical studies indicate that disruptions in the communication between muscle and other tissues and organs (such as bone, fat, and liver) play a crucial role in the development and progression of sarcopenia [83,84,85,86,87]. This muscle–organ interaction is mediated by tissue-specific molecules (e.g., myokines, osteokines, adipokines, hepatokines), which influence various muscle-wasting processes through autocrine, paracrine, and endocrine signaling pathways [88]. However, the specific role of individual biomolecules in the pathogenesis of sarcopenia remains unclear.

Common pathophysiological pathways, including low-grade chronic inflammation [81,89], mitochondrial dysfunction [90,91], oxidative stress [92,93], insulin and anabolic resistance [94,95], and other hormonal changes [96,97,98], may help explain the onset and progression of this deteriorating condition. Intervention trials have indicated that some of these pathological processes may be mitigated through proper nutrition and exercise [99,100,101].

## 4. Evidence of the Effect of Adherence to the Mediterranean Diet on Incident Sarcopenia

There are several systematic [31,102,103] and narrative reviews [79,104,105,106] that have examined the relationship between dietary patterns and sarcopenia—including the Mediterranean diet, with some of them also describing single foods or combining various healthy dietary patterns into a single category—and considered all types of studies (cross-sectional and longitudinal). Other reviews specifically addressed the role of dietary patterns in sarcopenia related to specific pathological conditions, e.g., cancer [107] or diabetes [108]. Only a few former reviews were focused specifically on the Mediterranean diet: a narrative review by McClure et al. in 2017 [109], two systematic reviews by Craig et al. in 2017 [32] and by Papadopoulou et al. in 2023 [110], and two systematic reviews and meta-analyses by Silva et al. in 2018 [40] and by Coelho et al. in 2021 [39] (Table 1).

We aimed to review the available evidence of the associations between adherence to the Mediterranean diet and incident sarcopenia or its components in prospective, longitudinal studies with at least a one-year follow-up. A thorough review of the literature published in PubMed from database inception to 1 December 2024 was conducted to identify articles on the Mediterranean Diet and sarcopenia as well as its components in the different definitions utilizing the following terms: “Mediterranean”, “diet”, “dietary patterns”, “sarcopenia”, “muscle weakness”, “muscle strength”, “muscle atrophy”, “muscle mass”, “physical performance”, combined with keywords like “cohort”, “longitudinal”, “prospective”, “incident”, “trial”, “intervention”, “randomized”. We also supplemented the search with studies familiar to the authors and additional forward citation searches. Cross-sectional studies and dietary interventions like energy restriction, which may lead to muscle mass loss, were excluded. Additionally, we did not consider interventions specifically targeting the prevention of sarcopenic obesity (low muscle mass combined with high-fat mass). We also excluded other types of articles, such as case reports, case series, editorials, letters to the editor, comments, short reports, short communications, and perspectives. Priority was given to the largest studies and the most recent publications. Table 2 describes the characteristics of the longitudinal prospective studies included in this review specifically investigating associations between adherence to a Mediterranean dietary pattern and sarcopenia or its components [114,115,116,117,118,119,120,121,122].

## 5. Discussion

We found nine prospective longitudinal studies with follow-ups of at least one year; despite the variability in the populations, both in terms of number and country of origin, and the differences in the methods used to assess adherence to the Mediterranean diet and sarcopenia or its components, the majority reported some significantly positive results (studies indicated in light blue in Table 2) [114,115,116,118,119,120,122]. Only two studies reported fully negative results (indicated in white in Table 2) [117,121]. The nine studies reviewed provided data from a total of 15,986 participants on the association between the Mediterranean diet and incident sarcopenia or surrogates of it. Most of the studies included both men and women with only one study including only women [118] and two studies including only men [119,120]. As shown in the table, the studies were conducted in diverse parts of the world: three studies were conducted in Italy [114,116,122], and one in the USA [115], China [117], Finland [118], Sweden [119], Australia [120], and Japan [121]. This illustrates the interest in studying this high-quality pattern in places other than the Mediterranean basin. There have been no clinical trials so far on this topic, which makes it difficult to establish a causal link with certainty. Most studies included older adults, and the most commonly used method to evaluate adherence to the Mediterranean diet, among others, was the score developed by Trichopoulou et al. [113] or adaptations of it. The methods for evaluating sarcopenia were highly variable. Four studies used the definitions of sarcopenia given by the EWGSOP [118,119,122] and AWGS [117], while the remaining assessed muscle mass, muscle strength, physical performance, or their combinations with diverse methodologies.

The substantial heterogeneity in the methods used to define the presence of sarcopenia and scores assessing adherence to the Mediterranean diet may also help explain the generally negative results of the systematic reviews and meta-analyses of cohort and cross-sectional studies shown in Table 1. This does not allow us to definitively conclude that there is or there is not an association between adherence to the Mediterranean diet and incident sarcopenia or its components, as was observed in most of the prospective longitudinal studies we reviewed (Table 2). However, it is important to acknowledge that these results may stem more from methodological flaws than from an inherent negativity in the outcomes themselves.

Among the longitudinal prospective studies reviewed (Table 2), adherence to the Mediterranean diet had a positive impact on muscle mass in two studies [118,120], on walking speed in three studies [115,116,118], and on physical performance, assessed with a short physical performance battery, in one study [114], although no studies showed an association with handgrip strength. Only one study reported a clear association of lower adherence to the Mediterranean diet with a significantly higher prevalence of probable sarcopenia [122]. Another study [119] indirectly found this association: a significant inverse association with sarcopenia was observed for each standard deviation increase in the Mediterranean diet score of adherence, and this relationship persisted after adjusting for potential confounders [119]. The lack of association in other studies may be explained, again, by variations in the definition of sarcopenia, which are substantial. Indeed, the prevalence of sarcopenia can be very different depending on the method used to assess it [17]. A recent analysis of the DO-HEALTH study among community-dwelling participants aged over 70 years from four European countries examined 12 different sarcopenia definitions and found highly variable prevalence of sarcopenia ranging between 0.7% using the EWGSOP2 or AWGS2 definitions up to 16.8% using the Delmonico definition. Furthermore, most sarcopenia definitions did not overlap when identifying sarcopenia on an individual participant-level [17]. These significant discrepancies in the prevalence of sarcopenia indicate that the concept of sarcopenia may require reconsideration in order to accurately and reliably identify individuals with impaired muscle health. The absence of a single, standardized definition of sarcopenia has led to several challenges, including the fact that results from research into the prevalence, incidence, causes, and consequences of sarcopenia are often difficult to reconcile, as varying definitions can result in significantly different estimates. This may have clinical implications, as healthcare providers may be uncertain about which measures or cut-off points to use when assessing patients. The absence of a standardized definition may also hinder the development of validated and specific clinical interventions for sarcopenia. In this regard, several study groups dedicated to sarcopenia involving experts from leading sarcopenia societies worldwide have established a collaborative initiative, The Global Leadership Initiative in Sarcopenia (GLIS), aiming to reconcile the various conflicting definitions of sarcopenia into a single, unified classification that would serve as the gold standard for assessing the condition through a comprehensive and transparent International Delphi Consensus process [123], which is expected to be available in the near future.

Sarcopenia is frequently considered a key factor in frailty and functional disability [124]. In the past ten years, sarcopenia and frailty have emerged as key concerns in geriatrics. Although there is some overlap between the two conditions, approximately one-third of persons with sarcopenia do not experience frailty, and conversely, not all frail persons have sarcopenia [125,126]. A key issue in this context is the potentially unproductive debate over the causal relationship between the two. Determining whether frailty results from sarcopenia, or if sarcopenia is simply a clinical manifestation of frailty, would require significant effort and, from a practical perspective, is similar to the “chicken and egg” dilemma. However, it is interesting to note how, contrary to the little evidence of a relationship between adherence to the Mediterranean diet and the incidence of sarcopenia found in the present review, the results are convincing for the relationship of greater adherence to this healthy dietary and lifestyle pattern with a lower incidence of frailty reported in a recent meta-analysis [33]. Of note, in this meta-analysis most of the high-quality studies included used the Mediterranean diet adherence score described by Trichopoulou et al. [113] or scores based on it with minor adaptations, and the majority used the frailty index proposed by Fried et al. It is possible that by harmonizing the definitions used, more certain conclusions can be reached, and the recent proposal of the GLIS may play a role in this [123].

**Figure 3:**
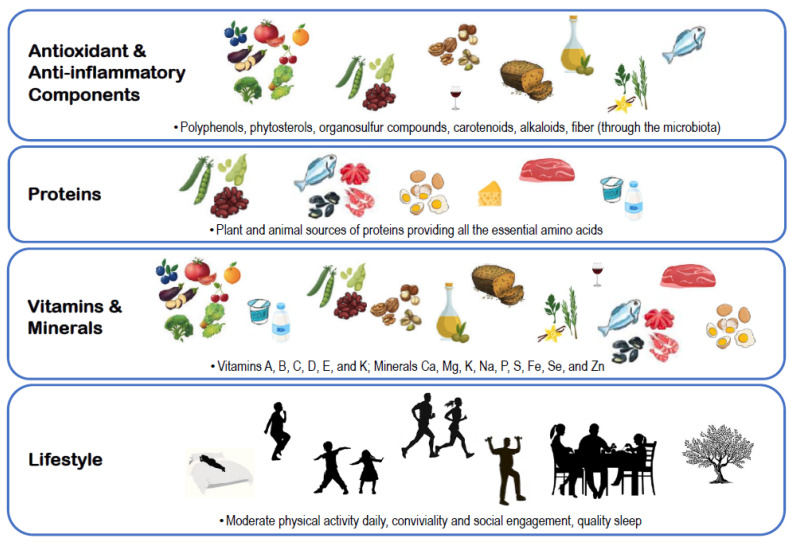
Components of the Mediterranean diet and lifestyle that may help preserve muscle mass and function.

Healthy high-quality dietary patterns, including the Mediterranean diet, are predominantly “plant-based,” emphasizing the consumption of plant-derived protein, high consumption of vegetables and fruits, nuts, and seeds, and the daily use of olive oil as the primary fat source. These diets also limit red/processed meat and processed foods [127] (Figure 1). The bioactive compounds contained in the nutritional components of the Mediterranean diet, such as polyphenols, polyunsaturated fatty acids, phytosterols, organosulfur compounds, carotenoids, and alkaloids, as well as fiber (through maintaining a balanced and healthy microbiota) (Figure 3), provide anti-inflammatory and antioxidant benefits [37,54] that help protect against chronic degenerative diseases like cardiovascular disease [128], diabetes [129], dementia [130], and cancer [131]. By preventing these diseases, i.e., multimorbidity, these plant-based dietary patterns can help prevent sarcopenia. Recent analyses from a large representative sample of the English older adult population followed for over 12 years found that multimorbidity at baseline was associated with a significantly higher risk of incident sarcopenia [25].

The results of the present review indicating that adhering to a Mediterranean diet is associated with improved parameters of muscle mass and function, and with one study indicating its association with incident sarcopenia, may be explained, at least in part, by the muscle-protective effects of this diet stemming from its balanced content of vitamins (e.g., A, B, C, D, E and K), minerals (calcium, magnesium, potassium, sodium, phosphorus, selenium, iron, sulfur, zinc) [46], and various antioxidants [51,52,56], which reduce oxidative damage and chronic inflammation (Figure 3). Inflammation, whether acute or chronic, promotes muscle protein breakdown [132], inhibits protein synthesis [133], and impairs cellular processes like autophagy and muscle regeneration [134], contributing to reduced muscle mass and strength [135]. Polyphenols, mono- and polyunsaturated fatty acids, and dietary fibers help mitigate systemic inflammation by lowering the production of pro-inflammatory markers like C-reactive protein, interleukin-6, and fibrinogen, and by modulating the gut microbiota [54].

The Mediterranean diet is characterized by a low intake of animal protein, considering only infrequent consumption of red and processed meat while encouraging the consumption of proteins from vegetable sources, fermented dairy, and fish [37,45] (Figure 3). There is ongoing debate in the literature about the impact of protein sources on sarcopenia, as animal and plant proteins differ in digestibility and may have distinct effects on muscle protein synthesis [136]. Nevertheless, a meta-analysis found that animal and plant proteins had similar effects on lean mass and muscle strength [137]. Additionally, persons with low total dietary protein levels had lower appendicular lean mass and quadriceps strength, regardless of their dietary pattern [138]. Older adults should be encouraged to consume protein-rich meals around the time of their exercise sessions [139]. During periods of acute catabolism (such as bed rest due to illness) or when physical activity is not possible, higher protein intake plays a crucial role in preserving skeletal muscle [140].

Other dietary factors may help explain the observed associations. For instance, persons adhering to a healthy diet are more likely to have a healthier lifestyle, better social engagement [141], and active lifestyles [142], which may help reduce the age-related decline in muscle mass and function. Adhering to the Mediterranean diet and lifestyle, which includes strong social networks, regular physical activity, and good sleep quality, has also been linked to improved cognitive function and a lower risk of dementia [143,144] (Figure 3). This is particularly significant given that persons with dementia may be less adherent to the Mediterranean diet and are less physically active [145], and they may also frequently experience loss of independence, as well as frailty and sarcopenia [146].

## 6. Conclusions

In conclusion, adherence to the Mediterranean diet generally had a positive impact on muscle mass and physical performance in prospective longitudinal studies specifically designed for this purpose. The fact that only one of the studies reported an association of adherence to this high-quality healthy dietary pattern with sarcopenia may be explained, at least in part, by the heterogeneity of sarcopenia definitions and assessment tools. Future well-designed and sufficiently large intervention studies are needed to confirm the encouraging findings of the current observational evidence. Nevertheless, the Mediterranean diet, with its focus on nutrient-rich, anti-inflammatory, and muscle-supporting foods, as well as on a healthy lifestyle, should be promoted in order to help preserve muscle mass and function as persons age. When combined with regular physical activity, it may offer a powerful approach to preventing or delaying sarcopenia, helping to maintain strength, mobility, and quality of life in older adults.

## Figures and Tables

**Table 1:** Summary of results from narrative, systematic reviews and meta-analyses of cohort studies/cross-sectional studies specifically on association of Mediterranean diet with sarcopenia and its components.

Authors Country Year	Type of Review	N. and Type of Studies Included	N. of Participants (Range)	Age (Years)	MedDiet Adherence Assessment	Sarcopenia Assessment	Summary of Results
McClure et al. Australia 2017 [109]	NR	5 longitudinal cohorts and 7 cross-sectional	192 to 2983	≥60	MEDAS; MDS; MED; MSDPS; mMTD score; PCA/FFQ	WS; low physical activity; SMI; MM; SPPB; IHG; ALM/BMI	One longitudinal study showed that higher MedDiet adherence was associated with a faster WS. The other longitudinal studies did not include sarcopenia as an outcome. All 7 cross-sectional studies reported a positive association of adherence to the MedDiet with WS, SPPB, muscle mass, ALM/BMI and IHG.
Craig et al. UK 2017 [32]	SR	1 prospective cohort study on sarcopenia (other 2 on fracture incidence)	2948	≥65	MDS; PCA/FFQ; KIDMED index	AWGS algorithm	The only prospective study included found no association between MedDiet adherence and incident sarcopenia
Silva et al. Brazil 2018 [40]	SR and MA	8 prospective cohorts and 4 cross-sectional	192 to 3519	48 to 84	MDS; MED	AWGS algorithm; MM, strength, and power; physical function impairment (Rosow and Breslau scale; SF-12)	The cohort data indicated no association between adherence to MedDiet and sarcopenia; cross-sectional results showed a positive relationship.
Coelho et al. Brazil, Greece, Italy 2021 [39]	SR and MA	19 cross-sectional studies and 34 prospective studies	77 to 28,775	≥60	MEDAS; MDS; 55- and 13- point MedDiet scores; aMED; Australian MDS; MSDPS; PCA/FFQ	IHG; TUG; 6MWT; FSST; BBS; WS	High MedDiet adherence was cross-sectionally associated with better WS and knee muscle strength speed. In the prospective studies, the pooled analysis indicated no significant associations with IHS and mobility.
Papadopoulou et al. Greece 2023 [110]	SR	4 cross-sectional, and 6 prospective studies	90 to 3675	≥65	MEDAS; MDS; MED; MSDPS; mMTD score; PCA/FFQ	WS; EWGSOP2; AWGS algorithm; 20 m walking test; sitting time; SARC-F; SARC-CalF; SPPB; IHG; ALM	Only 3 studies assessed sarcopenia and 4 measured MM. MedDiet adherence had, in general, a positive role in MM and muscle function, while the results were less clear for muscle strength. There was no evidence of a positive effect of the MedDiet on sarcopenia.

ALM: appendicular lean mass; aMED: Mediterranean diet proposed by Panagiotakos et al. [111]; AWGS: Asian working group on sarcopenia; BBS: Berg balance scale; BMI: body mass index; EWGSOP2: European working group on sarcopenia in older people; FFQ: food frequency questionnaire; FSST: four square step test; IHG: isometric handgrip strength; KIDMED: children and adolescents based on a Mediterranean diet quality index tool developed by Serra-Majem et al. [112]; MA: meta-analysis; MDS: Mediterranean diet score developed by Trichopoulou et al. [113]; MED: alternate mediterranean food score; MEDAS: Mediterranean diet screener; MedDiet: Mediterranean diet; mMTD: modified Mediterranean-type diet; MM: muscle mass; MSDPS: Mediterranean-style dietary pattern score; PCA, principal component analysis; MWT: minute walking test; SMI: skeletal muscle index; SPPB: short physical performance battery; SR: systematic review; TUG: timed “up and go”; WS: walking speed.

**Table 2:** Summary of results from longitudinal prospective studies on association of Mediterranean diet and sarcopenia and its components.

Authors Country Year	Type of Study	N. of Participants	Age (Years)	Follow-Up (Years)	MedDiet Adherence Assessment	Sarcopenia Assessment	Summary of Results
Milaneschi et al., Italy 2011 [114]	Prospective	935—55.6% women	74.1 ± 6.8	3, 6, 9	MDS	SPPB	Higher MedDiet adherence was associated with less of a decline in SPPB.
Shahar et al., USA 2012 [115]	Prospective	2225—54.1% women	74.5 ± 2.8	8	MDS	20 m walking test	Higher MedDiet adherence was an independent predictor of less of a decline in usual 20 m WS.
Talegawkar et al., Italy 2012 [116]	Prospective	690—51.7% women	73 ± 6.24	6	MDS	HGS 15 ft (4.57 m) walking test	Higher MedDiet adherence was associated with lower risk of low WS.
Chan et al., China 2016 [117]	Prospective	6905—42.6% women	≥65	4	MDS	AWGS algorithm	MedDiet (as well as other dietary patterns examined) was not associated with incident sarcopenia in men and women.
Isanejad et al., Finland 2017 [118]	Prospective	554—100% women	65 - 72	3	MDS	EWGSOP2	Higher MedDiet adherence was associated with faster WS, greater LBMQ, and better performance in squat tests.
Karlsson et al., Sweden 2020 [119]	Prospective	254—100% men	70.9 ± 0.6	16	Modified MDS	EWGSOP1	An inverse relationship with sarcopenia was found for each SD increment in the MDS (crude OR: 0.68; 95% CI 0.46–0.99), which remained after adjusting for potential confounders.
Cervo et al., Australia 2021 [120]	Prospective	794—100% men	81.1 ± 4.5	5	MEDI-LITE score	ALM/BMI WS IHS	MedDiet adherence was associated with higher ALM/BMI.
Huang et al., Japan 2021 [121]	Prospective	666—56.5% women	69.4 ± 4.4	3	Adapted MDS	SMI HGS WS	Adherence to MedDiet was not associated with changes in SMI, HGS, or WS.
Cacciatore et al., Italy 2023 [122]	Prospective	2963—54.4% women	72.8 ± 5.7	6	Modified MEDI-LITE score	EWGSOP2	Lower MedDiet adherence was associated with a significantly higher prevalence of probable sarcopenia (25.9%) vs. those with good (19.1%) or high (15.5%) adherence.

ALM: appendicular lean mass; AWGS: Asian working group on sarcopenia; BMI: body mass index; EWGSOP2: European working group on sarcopenia in older people; HGS: handgrip strength; IHG: isometric handgrip strength; LBMQ: lower body muscle quality; MDS: Mediterranean diet score developed by Trichopoulou et al. [113]; MedDiet: Mediterranean diet; MEDI-LITE: Mediterranean literature; SMI: skeletal mass index; SPPB: short physical performance battery; WS: walking speed.
